# Measuring time utilization of pharmacists in the Birmingham Free Clinic dispensary

**DOI:** 10.1186/s12913-016-1787-6

**Published:** 2016-09-29

**Authors:** Arielle M. Fisher, Michael Q. Ding, Harry Hochheiser, Gerald P. Douglas

**Affiliations:** 1 Center for Health Informatics for the Underserved, Department of Biomedical Informatics, School of Medicine, University of Pittsburgh, Pittsburgh, PA USA; 2 Intelligent Systems Program, University of Pittsburgh, Pittsburgh, PA USA

**Keywords:** Time and motion studies, Lean healthcare, Vulnerable populations, Workflow, Electronic health records, Clinical pharmacy information system, Medical informatics applications

## Abstract

**Background:**

Free and charitable clinics are a critical part of America’s healthcare safety net. Although informatics tools have the potential to mitigate many of the organizational and service-related challenges facing these clinics, little research attention has been paid to the workflows and potential impact of electronic systems in these settings. In previous work, we performed a qualitative investigation at a free clinic dispensary to identify workflow challenges that may be alleviated through introduction of informatics interventions. However, this earlier study did not quantify the magnitude of these challenges. Time-motion studies offer a precise standard in quantifying healthcare workers’ time expenditures on clinical activities, and can provide valuable insight into system specifications. These data, informed by a lean healthcare perspective, provide a quality improvement framework intended to maximize value and eliminate waste in inefficient workflow processes.

**Methods:**

We performed a continuous observation time-motion study in the Birmingham Free Clinic dispensary. Two researchers followed pharmacists over the course of three general clinic sessions and recorded the duration of specific tasks. Pharmacists were then asked to identify tasks as value-added or non-value-added to facilitate calculation of the value quotient, a metric used to determine a workflow’s level of efficiency.

**Results:**

Four high-level workflow categories occupied almost 95 % of pharmacist time: prescription (Rx) preparation (39.8 %), clinician interaction (21.5 %), EMR operations (14.8 %), and patient interaction (18.7 %). Pharmacists invested the largest portion of time in prescription preparation, with 21.8 % of pharmacist time spent handwriting medication labels. Based on value categorizations made by the pharmacists, the average value quotient was found to be 40.3 %, indicating that pharmacists spend more than half of their time completing tasks they consider to be non-value-added.

**Conclusions:**

Our results show that pharmacists spend a large portion of their time preparing prescriptions, primarily the handwritten labeling of medication bottles and documentation tasks, which is not an optimal utilization of pharmacist expertise. The value quotient further supports that there are many wasteful tasks that may benefit from workflow redesign and health information technology, which could result in efficiency improvements for pharmacists.

**Electronic supplementary material:**

The online version of this article (doi:10.1186/s12913-016-1787-6) contains supplementary material, which is available to authorized users.

## Background

Free and charitable clinics provide healthcare services to approximately 6 million people, representing a critical part of the healthcare safety net system in the United States [1]. These nonprofit organizations offer services to the underserved, uninsured, and working poor for free or with nominal charges. An increasing burden of chronic care in conjunction with a diverse patient population result in organizational and service-related challenges for low-resource clinics, which are understudied work environments in healthcare [2]. To alleviate some of these challenges, some free clinics have recently adopted electronic systems to improve the provision of care while saving valuable staff time [3]. These systems may include bespoke or custom databases and spreadsheets, patient management systems, and full-fledged electronic medical records (EMRs) [3]. While this may be appealing to low-resource clinics, many of these systems are not designed to support workflows unique to these clinics, and there are limited data regarding their impact on staff productivity and time-utilization in this setting.

Informatics tools have immense potential to assist healthcare workers and improve productivity if they are designed to support user workflow [4]. Designing a problem-driven informatics intervention to improve productivity requires a detailed understanding of user workflow and clinical processes, and more importantly the challenges users encounter. Identifying workflow challenges to inform technological design is a complex task, and many qualitative methods such as interviews and focus groups rely on the user’s ability to clearly articulate his/her tasks and needs. Time-motion studies in which an observer follows a subject and continually records the duration of every activity in a data collection tool can provide quantitative validation to qualitative investigations [5].

An increase in adoption of informatics and information technology (IT) systems in healthcare has prompted the need to study and evaluate the adoption of such systems and how they impact the quality, efficiency and costs of healthcare [6]. Time-motion studies are business efficiency techniques that have been adopted in the biomedical domain to help address these evaluation needs [6]. For example, time-motion studies have been used to measure the effect of an electronic health record on physician time utilization, in addition to identifying the main drivers of inefficiency in the nursing work process [7,8]. Using time-motion observations to quantify healthcare workers’ time expenditures on different clinical activities can provide valuable insight into system specifications and workflow redesign [7]. Enhancing process efficiency may improve staff productivity and related organizational challenges.

Lean is a methodology used to continuously improve process quality and efficiency by eliminating waste [9]. Waste can be defined as anything that does not add value in the eyes of the customer [9]. The core of lean involves determining the value of any given process, which is identified by the customer, distinguishing value-added steps from non-value-added steps (a process called “value stream mapping”), and eliminating waste so that every step ultimately adds value to the process [10–12]. Lean describes primary and internal processes. Primary processes serve the external customer, such as patients and their families, while internal processes serve healthcare staff and other internal customers, such as hospitals and insurers, in support of the primary processes [9,12]. Primary processes are typically easier to see, particularly in healthcare, however internal processes are necessary to create value in the primary process. Simply put, lean aims to maximize value and eliminate waste, and has been successfully implemented in various healthcare settings (e.g. operating rooms, hospital pharmacies) resulting in cost savings, quality and safety improvements, and higher patient and employee satisfaction [9–12].

In this study, we utilized lean principles to improve the therapeutics value stream at the Birmingham Free Clinic (BFC) in Pittsburgh, PA. The BFC is a free, walk-in clinic offering primary care and pharmaceutical services to a medically vulnerable population. An on-site dispensary enables access to essential medicines [13]. The introduction of an EMR at the BFC has improved several clinical processes. However, pharmacists report that the EMR has not directly improved their productivity, as it is unable to accommodate their workflow. While many of the clinic operations have become automated through the introduction of the EMR, the dispensary continues to maintain paper-based dispensing and inventory control processes. This hybrid EMR-paper system only exacerbates workflow inefficiencies and creates a boundary between the clinical and pharmaceutical services. [14].

Previously, we performed a qualitative investigation at the BFC dispensary to identify workflow inefficiencies that may be alleviated through introduction of an informatics intervention [14]. These inefficiencies included the process of handwriting medication labels, redundant documentation, and a lack of visibility into the current inventory during prescribing [14]. We proposed a framework for an informatics intervention that would streamline the dispensing process, reduce documentation, and automate inventory control [14]. However, this prior qualitative study did not provide any insight into the relative impact of relevant inefficiencies in the current workflow. To determine where to focus this intervention and maximize value, an understanding of the magnitude of the workflow challenges in the dispensary was necessary. We conducted a continuous observation time-motion study informed by a lean perspective to measure how pharmacists allocate their time, with the ultimate goal of reducing waste in non-value-added tasks.

## Methods

### Setting

The BFC utilizes a community-campus partnership to provide care to approximately 1,900 patients, with 3,000 patient encounters annually [13]. Volunteer pharmacists and students from the University of Pittsburgh School of Pharmacy work collaboratively with the medical team to provide in-depth medication therapy management (MTM) and disease state management for patients with chronic diseases. The dispensary provides on average 2–3 prescriptions per patient encounter, resulting in nearly 9,000 annual dispensations. Pharmacists also help patients apply for Patient Assistance Programs (PAPs), offered by most pharmaceutical manufacturing companies, to provide prescription drugs to low-income persons who lack medical insurance [15].

### Codebook development and pilot studies

An initial set of ten task subcategories was developed based on our prior qualitative analysis [14]. These task subcategories described pharmacist activities. We conducted a pilot study to test these codes and determine inter-rater reliability. Two researchers (AMF and MQD) simultaneously observed the same pharmacist during one three-hour session and recorded the time taken to accomplish these tasks. These data were not analyzed in the actual study. After the pilot session, the researchers were encouraged to ask questions about the appropriate categorization of pharmacist tasks to ensure completeness of the subcategories. We calculated Cohen’s kappa to measure inter-rater reliability [16].

Following the completion of the first pilot session, we modified our task subcategories and definitions so that they were more reflective of pharmacist activities at the BFC. We also clustered relevant subcategories into higher-level workflow categories. A second pilot session was performed with the improved coding system, allowing for additional familiarization with data collection. Both researchers observed the same pharmacist for three hours. Cohen’s kappa was recomputed to measure inter-rater agreement with the new coding system [16].

### Data collection

We collected data using the Time Motion Study application (Graphite Inc., http://www.graphiteinc.com/casestudy_mobileapps.aspx) for Android devices. This software allowed the observer to create a list of motions, i.e. task subcategories, to track pharmacist activity. Timing began as soon as the task was selected and ended when the observer selected a new task.

Each researcher observed a different pharmacist during a given data collection session. Pharmacists often work in pairs at the BFC. Data was collected to reflect the entire shift of the pharmacist, from when the pharmacist started working to when he/she left the clinic. Thus, data collection began approximately 30 min before the first patient appointment for that day. This allowed the observers to collect pre-work activities completed by the pharmacists, such as patient documentation and inventory maintenance. Data collection continued through general care hours until onsite care was completed for the last scheduled patient of that clinic session, in addition to any post-work activities. Each session lasted roughly three hours.

### Statistical analyses

The main objective of our study was to measure how much time pharmacists spend on different tasks in their current workflow. First, we measured the amount of time pharmacists spend in each higher-level workflow category (e.g. Rx preparation, patient interaction) for the entire dataset. These data helped us identify which main categories consume the largest proportion of pharmacist time. We then measured the pharmacists’ time distribution in each of the task subcategories. Data was recorded in the application in comma separated value files. RStudio 0.98 (RStudio Inc., www.rstudio.com) and R 3.1 were used for analysis. We created bar charts to help visualize these data.

### Value stream mapping

To maximize value by eliminating redundancy and waste, it is necessary to classify the task subcategories as value-added or non-value-added activities, thereby identifying potential areas for improvement. In lean language, this is the value stream mapping phase, and requires identification of value as perceived by the customer [9]. We identified the BFC pharmacists as the internal customer in this study. We recognize that there are many customers affected by the therapeutics value stream, and that the patient represents the ultimate external customer. However, it is the pharmacist who is immediately affected by the internal processes captured in this study, and therefore satisfying their needs is our focus. The pharmacists specified efficiency as the value they desired, which is defined as being able to produce something with the minimum amount of time, motion, and resources. We asked the pharmacists to identify tasks as non-value-added and value-added in their current state, with the ultimate goal of reducing or eliminating waste embedded in each task.

We used the value categorizations indicated by the BFC pharmacists to calculate the value quotient for each dataset. The value quotient is a metric for determining the efficiency of a workflow in meeting patient needs while effectively using the resources he/she has been granted [17]. This calculation provides insight into the amount of time pharmacists spend completing tasks they consider to be non-value-added, indicating time that might be redirected to more patient-centered tasks such as providing education on proper medication usage. Our goal is to increase the value quotient by decreasing the amount of time invested in these non-value-added tasks. The value quotient formula is shown below:

$$ \mathbf{Value}\ \mathbf{Quotient}\ :\ \mathrm{value} - \mathrm{added}\ \mathrm{time}/\mathrm{total}\ \mathrm{time} $$

The numerator was calculated by summing the total time spent performing value-added tasks for the entire dataset. The denominator represents the total time spent performing all tasks.

## Results

Two observers (AMF and MQD) collected time-motion data during three independent clinic sessions between September and November 2014. Three different pharmacists were observed over the course of these time-motion sessions for a combined total of approximately 16.5 h. Cohen’s kappa for the first pilot session was found to be κ = 0.806, indicating strong agreement between raters. We made several changes to the coding system after this first pilot session: two subcategories were added for completeness (consulting clinician and traveling); the definition of dispensing medication was broadened to accommodate a multiple-step process; and strict initiation and termination times were defined for each task subcategory. The final codebook is shown in Table 1 and a complete list of the task subcategories, definitions, value categorizations, and their initiation/termination protocols can be found in Additional file 1: Table S1. After modifying and finalizing the codebook, Cohen’s kappa was calculated to be *κ* = 0.808 for the second pilot session, confirming that agreement between raters remained strong.

**Table 1:** Task subcategories and categories used in data collection

Pharmacist task category	Pharmacist task subcategory
Prescription (Rx) Preparation	Hunting for medication
	**Traveling**
	Dispensing medication
	Labeling medication bottles
	Duplicate documenting
Clinician Interaction	**Consulting clinician**
	Teaching
Patient Interaction	Counseling patients
	Patient Assistance Program application initiation
	Patient Assistance Program discussion of pending forms
EMR Operations	EMR Operations
Other	Other

Task subcategories traveling and consulting clinician were added after the first pilot session. These items are indicated in bold. *EMR* Electronic Medical Record

**Fig. 1:**
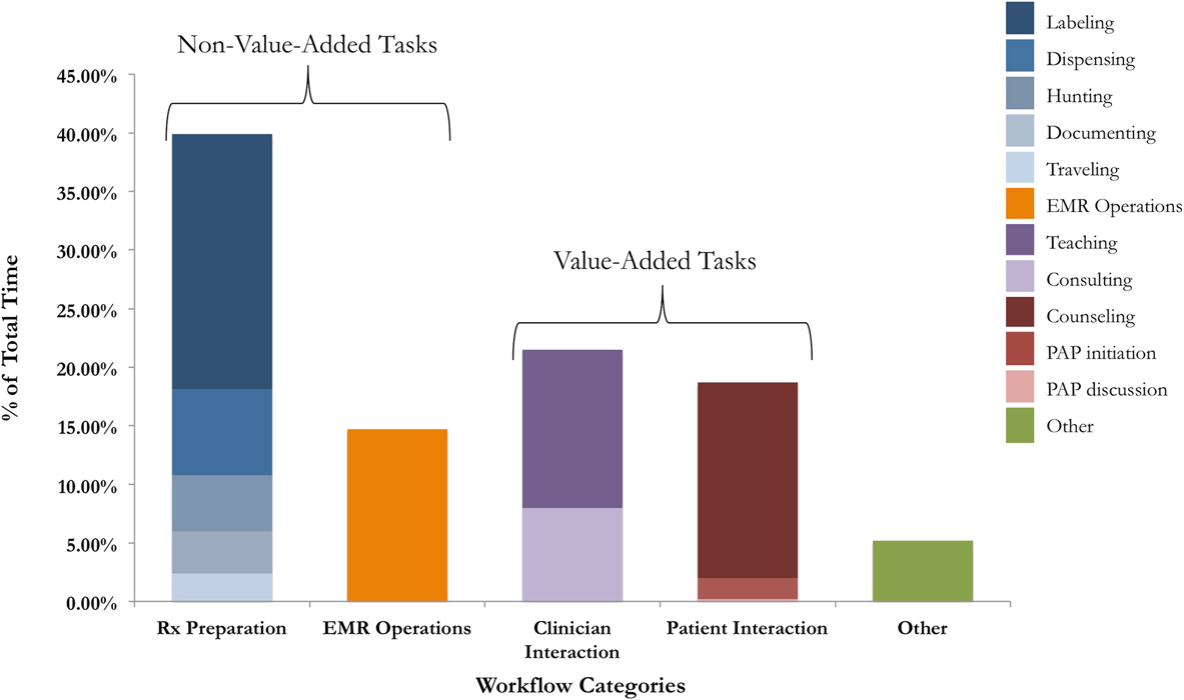
Percentage of total time investment. Proportion of time spent in each major workflow category, comprised of associated task subcategories. For the purposes of this diagram, EMR Operations is classified as a non-value-added task

We compare pharmacist time investment between the four main workflow categories described in Table 1: Rx preparation, clinician interaction, patient interaction, and EMR operations. Pharmacist time utilization by each task subcategory is shown in the stacked bar diagram in Fig. 1, where the four main workflow categories are deconstructed into their associated subcategories in each bar for the entire dataset (16.5 h).

### Prescription preparation

Pharmacists invest 39.8 % of their time into preparing prescriptions for dispensation. This category includes five task subcategories: traveling (2.3 %), duplicate documenting (3.6 %), hunting for medication in inventory cabinets (4.8 %), dispensing medication (7.3 %), and handwriting medication labels (21.8 %). The pharmacists identified these tasks as non-value-added, or potential areas for improvement, as they are inefficient and labor-intensive. While they are necessary to fill prescriptions given the resource constraints of the BFC, redundant documentation and handwriting medication labels require significant time investment and attention to detail, in addition to underutilizing pharmacist expertise.

### EMR operations

Our results indicate that pharmacists spend 14.8 % of their time using the EMR. These activities include reviewing patient demographic and allergy information, past and current medication orders, and modifying orders entered by the prescriber during examination. The pharmacists report that the EMR does not directly benefit their workflow efficiency, as the computerized physician order entry (CPOE) correction process is quite cumbersome. While they recognize that current use of the EMR is both necessary and productive for clinical care, it often creates redundant work when combined with their paper-based dispensing process. It was difficult for the observers to differentiate value-added versus non-value-added use of the EMR without interrupting the pharmacist, nor was this the scope of our study. For this reason, the value quotient was calculated twice for each dataset, once with this task considered to be value-added and once with it classified as non-value-added.

### Clinician interaction

Clinician interaction consumes more than a fifth (21.5 %) of pharmacist time. This category includes clinician consultation (8 %) and teaching students and/or other clinician volunteers (13.5 %). Due to the limited formulary at the BFC, the attending physicians often consult with the pharmacist to determine appropriate and affordable treatment plans. This consultation also provides a teaching opportunity for clinicians and students alike, which is a valuable component of the collaborative model at the BFC. All tasks in this category add direct value to both patient care and student education, as indicated by the pharmacists.

### Patient interaction

Pharmacists spend 18.7 % of their total time interacting with patients, which includes direct pharmacist-patient counseling (16.7 %) and initiation and discussion of PAP applications (1.8 % and 0.2 %, respectively). Initiation of a PAP application occurs when a patient needs a prescription medication but lacks drug insurance. This process begins in the dispensary with a pharmacist assisting the patient in filling out a paper application. If a patient has already initiated a PAP application in a previous visit, the pharmacist often discusses the application status with the patient during counseling. These discussions tend to occur if a patient has failed to bring in required income documentation for completion of the PAP application. The pharmacists identified all tasks in the patient interaction category as value-added as they are integral to patient education and chronic disease state management.

### Other

Tasks completely unrelated to any aspect of the pharmacist’s job, such as casual conversation, using the restroom, or filling a water bottle, consume 5.2 % of pharmacist time. These tasks will not be targeted by the lean intervention, and there is no clear way to classify them as value-added or non-value-added. For these reasons, they were not included in the value quotient calculations.

### Value quotient

Based on the value categorizations made by the pharmacists, the value quotient range for the entire dataset was found to be 40.3 %– 54.8 %. The lower bound represents the value quotient when the EMR was considered to be a non-value-added task while the higher bound considers EMR operations to be a value-added task. All value quotients are shown in Table 2.

**Table 2:** Value quotients for each dataset

Session	Value Quotient (%) [EMR = non-value-added]	Value Quotient (%) [EMR = value-added]
1	39.3	56.5
2	43.6	51.4
3	38.0	56.5
Overall	40.3	54.8

Value quotient is calculated by dividing the time spent on value-added tasks by the total time. We calculated two value quotients for each dataset: 1) EMR Operations as a non-value-added category; 2) EMR Operations as a value-added category

## Discussion

This continuous observation time-motion study provides insight into the actual time distribution of pharmacist activities at the BFC. The results do not rely on reporting by the pharmacists but rather on how their time is actually allocated to different tasks. Our findings demonstrate that pharmacists spend nearly 40 % of their time preparing prescriptions for dispensing, which is a collection of necessary tasks they consider to be inefficient and labor-intensive. The value quotient indicates that pharmacists invest more than half of their time performing tasks they consider to be non-value-added. These tasks may benefit from workflow redesign. By reducing waste in these tasks, pharmacists can redirect this time to focus on more patient-centered tasks, such as education and counseling.

Quantifying pharmacists’ time expenditures on different dispensing tasks in a low-resource setting is an understudied work area in United States healthcare. Specific comparisons across different studies are difficult to make due to variability in the implementation and reporting of time-motion methods, which may result in differences in the duration of observations, number of tasks, and reliability assessments [6]. Our results are somewhat comparable to those of a direct observation time-motion study for pharmacists’ activities in a 60-bed geriatric hospital in Malta, which found that pharmacists spend approximately 20.92 % of their time in the medication order category. This category includes the organization and delivery of medication to wards, in addition to several documentation tasks [18]. These activities are classified as administrative, meaning that they may be performed by competent non-pharmacist personnel [18]. While these activities are important, they should not consume large amounts of pharmacist time, which is better spent on clinical activities more closely directed toward individual patient care [18].

Results from our analyses suggest a similar conclusion, while doubling the amount of time spent on similar tasks (i.e. prescription preparation) compared to Wirth et al. [18]. Clinical pharmacists at the BFC currently spend the majority of their time on activities some hospital pharmacists consider to be administrative, which is a poor utilization of pharmacist expertise. The role of a clinical pharmacist in community pharmacies is to identify and resolve medication problems, such as prescribing errors, and problems that develop from patient behavior [19]. Clinical pharmacists have the knowledge and skill base to contribute to improved medication safety and effectiveness through collaborative participation in patient-specific medication and disease management [19]. These activities are critical in providing quality healthcare at the BFC, and require the majority of pharmacist time, attention, and skill.

While we recognize that process inefficiencies at the BFC exist due, in part, to their hybrid EMR-paper system, the magnitude of this inefficiency and its impact on high value tasks is surprising. The results from this study act as a data triangulation component to the workflow challenges we uncovered in our prior qualitative inquiry [14]. We found that medication labeling, triple documenting, and lack of inventory control were among the most significant workflow challenges [14]. Handwriting individual medication labels and redundant documentation tasks are time-consuming, labor-intensive, and prone to human error. Lack of inventory management results in unnecessary travel and search for medications, both time consuming tasks for BFC pharmacists. These tasks represent the main drivers of inefficiency and are amenable to workflow improvements. Focusing an informatics intervention on alleviating challenges associated with these tasks, such as computer-generated labels and automated inventory reduction, may reduce the amount of time pharmacists invest in their completion.

Lean principles have provided us with greater insight during analysis, but we recognize that this methodology may cause confusion regarding the pharmacists’ categorization of value-added and non-value-added tasks. In the current workflow, all tasks are necessary to dispense medication and counsel patients. Thus, one may argue that all tasks are value-added. This is particularly notable in the classification of dispensing, which consists of counting pills, as a non-value-added task. We understand that this classification may be too coarse given the nature of this task, and that further analysis may be necessary to understand what constitutes good use of pharmacist time in this setting. However, it is important to note that lean methodology encourages identification of value according to the customer, which explains our use of the value categorizations made by the pharmacists.

It is difficult to assess the generalizability of our findings. The pharmacists observed in this study have more than seven years of experience at the BFC. These pharmacists often used workarounds to optimize the amount of time spent on different tasks, minimizing their time investment on non-value-added tasks. We recognize that introducing new volunteers into the workflow is common practice for free clinics, and that these volunteers may take longer to complete the same tasks. Measuring the variability between pharmacists would be a useful study, yet this would be difficult at the BFC because the pharmacists do not evenly divide task load. Our data maintains consistency across clinic sessions and measures time investment of the most experienced BFC pharmacists. Thus, the calculated value quotients may be an overestimate, because less experienced pharmacists are likely to spend more time on non-value-added tasks due to not having had the opportunity to develop efficiency workarounds. We acknowledge that the Hawthorne effect may have also contributed to biased results, as it is possible pharmacists behaved differently during observations.

## Conclusions

The clinical pharmacists at the BFC play a critical role in providing patient care that extends beyond just dispensing medication. While documentation and other administrative activities are necessary, it is important these tasks are completed efficiently so the pharmacist can contribute fully to individual patient care. Our results indicate that pharmacists at the BFC allocate a large proportion of their time to prescription preparation, primarily the handwritten labeling of medication bottles and documentation tasks, which is not an optimal utilization of pharmacist expertise. The value quotient further supports the conclusion that pharmacists devote more than half of their time to tasks that are amenable to efficiency improvements. We believe that changes to the process and technology of medication management, documentation, and inventory control through introduction of a problem-driven informatics intervention may improve pharmacist efficiency and the safe delivery of quality patient care in this low-resource dispensary.

## Additional file

Additional file 1: Table S1. Complete list of task subcategories, their definitions, initiation/termination protocol, and value categorizations (DOCX 75 kb)

## Abbreviations

IT: Information technology
BFC: Birmingham Free Clinic
EMR: Electronic medical record
MTM: Medication therapy management
PAP: Patient Assistance Programs
CPOE: Computerized physician order entry
