# Supplementary material for: Measuring time utilization of pharmacists in the Birmingham Free Clinic dispensary
Source: BMC Health Serv Res. 2016 Sep 29;16:529. doi: 10.1186/s12913-016-1787-6 (PMC5043635; doi:10.1186/s12913-016-1787-6)
Supplement: Additional file 1: Table S1. — Complete list of task subcategories, their definitions, initiation/termination protocol, and value categorizations (DOCX 75 kb) [file 12913_2016_1787_MOESM1_ESM.docx]

| **Application**  **Abbreviation** | **Task**  **Subcategory** | | **Definition** | **Value definition** |
| --- | --- | --- | --- | --- |
| Hunting | Hunting for medication | | The physical search for stock medication in the cabinets.  **Begin:** When pharmacist slides open cabinet door to initiate search.  **End:** When pharmacist closes the door and/or found the medication. | Non-value-added |
| Labeling | Labeling medication bottles | | The manual process of writing individual medication labels for patient bottles.  **Begin:** When pharmacist starts writing on a label.  **End**: When pharmacist finishes taping the label on the bottle. | Non-value-added |
| Counseling | Counseling patients | | Explaining medication and administration instructions to patient during dispensation.  **Begin:** When pharmacist calls patient into the dispensary.  **End:** When patient exits the dispensary. | Value-added |
| EMR | EMR operations | | Any time spent in the EMR (i.e. entering & correcting orders, retrieving patient information, managing patient status).  **Begin:** When pharmacist turns to the computer and opens Epic.  **End:** When pharmacist isn’t actively using Epic. | Non-value-added / value-added |
| Documentation | | Duplicate documenting | Filling out the Pharmacy Activity Sheet with dispensation information.  **Begin:** When pharmacist starts writing patient information on PAS.  **End:** When pharmacist completes the document. | Non-value-added |
| Clinician | Consulting clinician | | Consulting physician to discuss patient treatment plans or answer questions regarding formulary.  **Begin:** When physician physically enters the dispensary and/or when pharmacist talks to physician in exam room.  **End**: When physician leaves dispensary and/or when pharmacist finishes conversation with physician. | Value-added |
| Papinit | PAP initiation | | Deciding to initiate a PMAP program with patient and beginning application process.  **Begin:** When pharmacist starts filling out application.  **End:** When pharmacist finishes working on application. | Value-added |
| Papdis | PAP discussion | | Talking to patient about bringing in missing application materials.  **Begin:** When pharmacist brings up PMAP during counseling.  **End:** When they stop talking about the application. | Value-added |
| Teaching | Teaching students/clinician volunteers | | Teaching sessions with pharmacy students and new volunteers.  **Begin:** When pharmacist initiates teaching session by asking questions of students.  **End:** When the conversation ends. | Value-added |
| Dispensing | Dispensing medication | | Retrieving empty medication bottles, counting pills, and filtering medication into bottles.  **Begin:** When pharmacist either begins counting pills or reaches for an empty medication bottles.  **End**: When pills are filtered into bottle. | Non-value-added |
| Traveling | Traveling in clinic | | When pharmacist moves between locations in the entire clinic (i.e. walking to exam room) and in the dispensary alone.  **Begins:** when pharmacist gets up from the desk.  **Ends:** When pharmacist returns. | Non-value-added |
| Other | Other | | Any arbitrary tasks that are unrelated to work tasks. For example, casual conversation or using the restroom. | Non-value-added (not included in value quotient calculation) |
